# Validation of Intravoxel Incoherent Motion MRI Using Perfused Explanted Human Livers

**DOI:** 10.1002/mrm.70184

**Published:** 2025-11-14

**Authors:** Gregory Simchick, James Rice, Leah M. Gober, Daniel Rice, Jennifer Philip, Alejandro Roldan‐Alzate, Diego Hernando

**Affiliations:** ^1^ Radiology University of Wisconsin‐Madison Madison Wisconsin USA; ^2^ Mechanical Engineering University of Wisconsin‐Madison Madison Wisconsin USA; ^3^ Surgery University of Wisconsin‐Madison Madison Wisconsin USA; ^4^ Medical Physics University of Wisconsin‐Madison Madison Wisconsin USA

**Keywords:** abdomen, deceased, diffusion weighted imaging, donor, ex vivo, intravoxel incoherent motion, liver, quantitative magnetic resonance imaging

## Abstract

**Purpose:**

Evaluate the feasibility of using perfused explanted human livers for validating intravoxel incoherent motion (IVIM).

**Methods:**

Eight (*n* = 8) explanted livers from deceased donors were obtained. The portal vein and hepatic artery of each explanted liver were connected to a perfusion system. IVIM data were acquired at four total volumetric flow rates (0, 0.6, 0.9, and 1.2 L/min). For each IVIM dataset, diffusion coefficient (*D*), relaxation‐corrected perfusion signal fraction (*F*_*c*_), blood velocity SD (*V*_*b*_), and/or pseudo‐diffusion coefficient (D*) were estimated. Linear mixed‐effects modeling was performed to determine if the effect of applied flow, flow rate, and fibrosis stage on the estimated IVIM parameters was significant (*F*‐tests), while correcting for temporal effect(s). Liver fibrosis stages were obtained from clinical histology.

**Results:**

*D* was independent of applied flow and total volumetric flow rate (*p* ≥ 0.30). *F*_*c*_ was approximately zero when no flow was applied and a positive, non‐negligible value that was independent of flow rate with applied flow (*p* < 0.001 and ≥ 0.58 for applied flow and flow rate effects, respectively). *V*_*b*_ and D* were dependent on applied flow and flow rate (*p* ≤ 0.04 with flow rate effect size ≥ 0.69 mm/s per L/min and 25.7 × 10^−3^ mm^2^/s per L/min for *V*_*b*_ and D*, respectively). Significantly lower (*p* ≤ 0.01) *F*_*c*_, *V*_*b*_, and D* estimates were observed for livers with moderate‐to‐advanced fibrosis (stages F2‐4) compared to no‐to‐mild fibrosis (stages F0‐1) (effect sizes = −0.74%, −0.60 mm/s, and −19.8 × 10^−3^ mm^2^/s, respectively).

**Conclusion:**

Perfused explanted human livers from deceased donors may serve as biologically accurate systems for validation of quantitative IVIM techniques.

## 1 Introduction

Intravoxel incoherent motion (IVIM) is a promising technique for the evaluation of diffusion and microvascular flow. The potential of IVIM has been demonstrated in several organs, including the liver for staging fibrosis [1, 2, 3, 4, 5, 6, 7]. Recently, several studies have demonstrated the ability of IVIM to detect no‐to‐mild liver fibrosis (stages ≤ F1) from moderate‐to‐advanced fibrosis (stages ≥ F2) [4, 5, 6, 7]. However, several technical challenges, including physiologic motion, noise‐related fitting instability of IVIM data, and often unaccounted‐for dependencies in the IVIM signal, have led to poor precision (repeatability and reproducibility) and high measurement variability of IVIM parameters across studies [1, 2, 8, 9, 10, 11, 12]. These challenges have limited the generalizability and clinical utility of IVIM. Recent technical developments in the acquisition [8, 13, 14, 15, 16, 17], modeling [8, 13, 14, 18], and post‐processing [11, 19, 20, 21] of liver IVIM data have sought to address these challenges and have demonstrated improved precision, which may enable IVIM to realize its clinical potential.

Despite this progress, systematic validation of IVIM is still needed for developing reproducible quantitative IVIM methods and understanding the relationships between IVIM parameters and disease. However, systematic validation has been precluded by challenges associated with histologic validation of in vivo IVIM studies. Core biopsies needed for histology are invasive and suffer from sampling bias and variability [22, 23, 24, 25]. Further, histological markers, such as vessel density, only provide indirect measures of microvascular flow, unlike IVIM parameters which are directly sensitive to microvascular flow [26, 27, 28]. In vivo studies also lack known reference conditions associated with flow, such as intravoxel blood volume and vascular velocities or flow rates.

Alternatively, phantom studies may facilitate validation of IVIM using highly controlled external flow conditions [29, 30, 31, 32, 33]. IVIM phantoms are fundamentally difficult to fabricate due to the complex biological microstructure and microvasculature that is responsible for IVIM signal decay. Previous studies have designed IVIM phantoms using flow pumps to perfuse packed resin or gelatin microspheres [29, 30], compressed sponges [31, 32], or artificial microvascular networks made from melt‐spun sugar [33]. However, these phantoms have limitations, including not accurately mimicking biological microstructure and microvascular geometry, contributions from coherent flow on the MR signal, and/or not containing distinct structural and incoherent flow compartments (i.e., both the diffusion and incoherent flow components of IVIM signal depended on external flow conditions). Therefore, highly controlled phantoms or systems that more accurately mimic biological structure and in vivo IVIM signal behavior are needed.

Explanted animal or human organs with externally controlled vascular flow may address current IVIM histologic and phantom study limitations. Machine‐perfused explanted organs have been extensively used to study drug delivery and organ condition for transplantation [34, 35, 36]. Recently, several studies have used MR to evaluate perfused explanted hearts [37, 38, 39], kidneys [40, 41, 42, 43], and livers [44, 45, 46]. Using perfused explanted porcine livers, temperature‐induced ischemia has been evaluated using ADC [44], and dynamic susceptibility contrast MRI has been used to evaluate the homogeneity of machine‐induced tissue perfusion [46]. However, to the best of the authors' knowledge, no studies have used perfused explanted human livers to validate MRI methods.

The purpose of this work was to evaluate the feasibility of using perfused explanted human livers for validating IVIM. Additionally, a secondary purpose was to evaluate the potential of IVIM to differentiate livers with no‐to‐mild fibrosis (stages ≤ F1) from moderate‐to‐advanced fibrosis (stages ≥ F2) using perfused explanted livers.

## 2 Methods

### 2.1 Donors and Explanted Livers

Eight (*n* = 8) explanted livers from deceased donors where the liver was unable to be used for transplantation (Table 1) and for whom there was authorization for use of organs for research were obtained from the University of Wisconsin Organ and Tissue Donation (Madison, WI) for this Health Insurance Portability and Accountability Act‐compliant study. According to the United States Code of Federal Regulations, research involving deceased individuals is not human subjects research and does not require Institutional Review Board oversight. The University of Wisconsin Institutional Review Board reviewed this study and confirmed that it did not qualify as human subjects research.

**TABLE 1:** Donors and explanted livers.

Donor	Sex	Age (year)	BMI (kg/m^2^)	Fibrosis stage	Type of death	Cause of death	Reason not transplanted	Preservation time before imaging (h)
1	F	62	25.6	F0	DCD (WIT 22 min)	Stroke	Logistics	22.5
2	M	69	26.0	F0	DCD (WIT 71 min)	Head Trauma	Excessive WIT	70.1
3	M	62	29.1	F0	DCD (WIT 24 min)	Stroke	Logistics + Steatotic	29.0
4	F	59	26.3	F1	DBD (No WIT)	Stroke	Fibrotic	46.1
5	M	49	46.4	F2	DBD (No WIT)	Anoxia	Fibrotic	56.5
6	F	49	33.1	F3	DCD (WIT 27 min)	Head Trauma	Fibrotic + Steatotic	81.3
7	F	60	34.3	F4	DCD (WIT 24 min)	Stroke	Fibrotic	41.7
8	F	59	26.7	F4	DCD (WIT 18 min)	Stroke	Fibrotic	42.1

Abbreviations: BMI, body mass index; DBD, donation after brain death; DCD, death after circulatory death; WIT, warm ischemic time.

Livers were recovered using standard surgical recovery techniques [47] and preserved at 4°C in cold preservation solution (UW Solution, Organ Recovery Systems, Itasca, IL). Prior studies suggest organ transplant viability and integrity up to 72 h of cold storage and hepatocyte cellular viability up to 96 h [48, 49]. Therefore, to maximize research liver utilization and maintain tissue and cellular integrity, the goal was to connect each explanted liver to the experimental perfusion setup and perform MR data acquisition (see the following two sections) within 96 h of preservation (i.e., time after aortic cross‐clamp and initialization of cold preservation). For each explanted liver, fibrosis stage was obtained from clinical histology of core biopsies, which was performed by the University of Wisconsin Organ and Tissue Donation service.

### 2.2 Perfusion Setup

**FIGURE 1:**
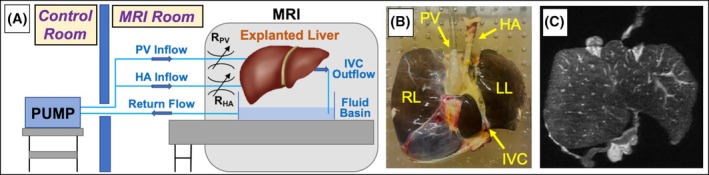
(A) Experimental MRI setup consisting of dual inflow lines for the portal vein (PV) and hepatic artery (HA). Using a peristaltic pump, organ preservation solution with constant, non‐pulsatile flow was perfused through an explanted liver. Each inflow line included a resistive valve (R) to constrain the inflow ratio of the PV versus HA. The inferior vena cava (IVC) was cannulated to facilitate extraction of the mixed outlet flow, which was collected in a fluid basin for return flow to the pump. (B) Image of the explanted liver and flow connections. (C) T2‐weighted image of the explanted liver. LL, left lobe; RL, right lobe.

Perfusion setup was performed at room temperature where explanted livers and the preservation solution were allowed to naturally warm from the preservation temperature. Dual inflow lines branching from a single peristaltic pump (Cole‐Parmer, Vernon Hills, IL) were connected to the portal vein and hepatic artery via the celiac axis of each explanted liver for venous and arterial inflow, respectively (Figure 1). Using resistive elements, the inflow of organ preservation solution to the portal vein was constrained to be larger than that of the hepatic artery, mimicking in vivo conditions [50]. Mixed outlet flow was expelled from the inferior vena cava and collected in a fluid basin for return flow to the pump.

Prior to MRI (see next section), the solution was circulated at a total volumetric flow rate = 0.6 L/min for approximately 30 min to condition the organ (i.e., allow the flow in the liver vasculature to reach equilibrium, which was defined as less than ±20 mL/min variation in the total flow rate). To confirm equilibrium flow in each inflow line, the solution was then circulated at total flow rates = 0.6, 0.9 and 1.2 L/min, which included rates typically used for normothermic transplant viability assessment and observed in vivo [50, 51, 52], and measurements of venous and arterial flow rates were obtained. Flow rates were measured using a clamp‐on 2.4 MHz ultrasonic flowmeter and data acquisition module with a 5 kHz sampling rate (PXL Series/TS Series Module, Transonic Systems Inc., Ithaca, NY). Venous‐to‐arterial flow rate ratios (=venous divided by arterial flow rate) were compared across different total flow rates using paired sample *t*‐tests (significance level *α* = 0.05 used for all significance tests in this work) and across explanted livers with fibrosis stages F0‐1 and F2‐4 for each flow rate using two‐sample *t*‐tests. Total perfusion setup time, including surgical connection of inflow lines, conditioning the organ, and acquiring flow measurements, ranged from approximately 90–150 min across livers.

### 2.3 MR Acquisitions

At room temperature, each explanted liver was imaged at 3 T with a 30‐channel flexible anterior array coil and a 32‐channel embedded posterior array coil (Signa Premier with AIR and PA Coils, GE Healthcare, Waukesha, WI). Imaging was performed using constant, non‐pulsatile, total volumetric flow rates = 0, 0.6, 0.9, and 1.2 L/min applied sequentially. For flow = 0, data were acquired at the start (0_start_) and end (0_end_) of each MRI exam to evaluate potential changes that may have occurred during the exam.

#### 2.3.1 Preservation Solution

For each explanted liver, circulated organ preservation solution was collected in 25 mL cylindrical plastic vials immediately before and after each MRI exam. Vials from all livers were then imaged at room temperature using three sequences: (1) 2D multi‐TI fast‐spin‐echo sequence to measure *R*_1_ of the preservation solution (*R*_1ps_), (2) 2D multi‐TE spin‐echo sequence to measure *R*_2_ of the preservation solution (*R*_2ps_), and (3) monopolar IVIM acquisition with multiple *b*‐values (see Section 2.3.4 below for details) to measure the diffusion coefficient of the preservation solution (*D*_ps_). For the multi‐TI fast‐spin‐echo sequence, one TI was acquired per TR using the following acquisition parameters: TI = [50, 400, 800, 1200, 1600, 2000, 2400, 4000] ms, TR = 15 s, TE = 6.5 ms, resolution = 1.80 × 1.80 × 2.00 mm^3^, one slice, and echo train length = 8. For the multi‐TE spin‐echo sequence, one TE was acquired per TR using the following acquisition parameters: TEs = [15, 25, 35, 50, 75, 150, 300] ms, TR = 10 s, resolution = 1.95 × 1.95 × 10.0 mm^3^, and three slices with a 2 mm gap.

#### 2.3.2 Liver T1‐Weighted Imaging

At each flow rate, liver volume accelerated flex acquisition, which is a T1‐weighted 3D fast spoiled gradient‐echo sequence, was acquired for liver volume estimation using the following parameters: TI = 23 ms, TE = 1.52 ms, TR = 3.34 ms, resolution = 1.11 × 1.44 × 3.59 mm^3^, 60 slices, flip angle = 15 degrees, and bandwidth = 162.8 kHz.

#### 2.3.3 Liver STEAM‐MRS

Multi‐TE‐TR STEAM‐MRS was acquired at each flow rate from a voxel placed in the right lobe of the liver parenchyma (placed to avoid large vessels, bile ducts, and liver edge) using the following parameters: 32 spectra including 4 pre‐acquisitions, TRs = 140–1990 ms, TEs = 10–110 ms, mixing time = 5 ms, spectral width = ±2500 Hz, voxel size = 25 × 25 × 25 mm^3^, and 256 samples [53].

#### 2.3.4 Liver IVIM

**FIGURE 2:**
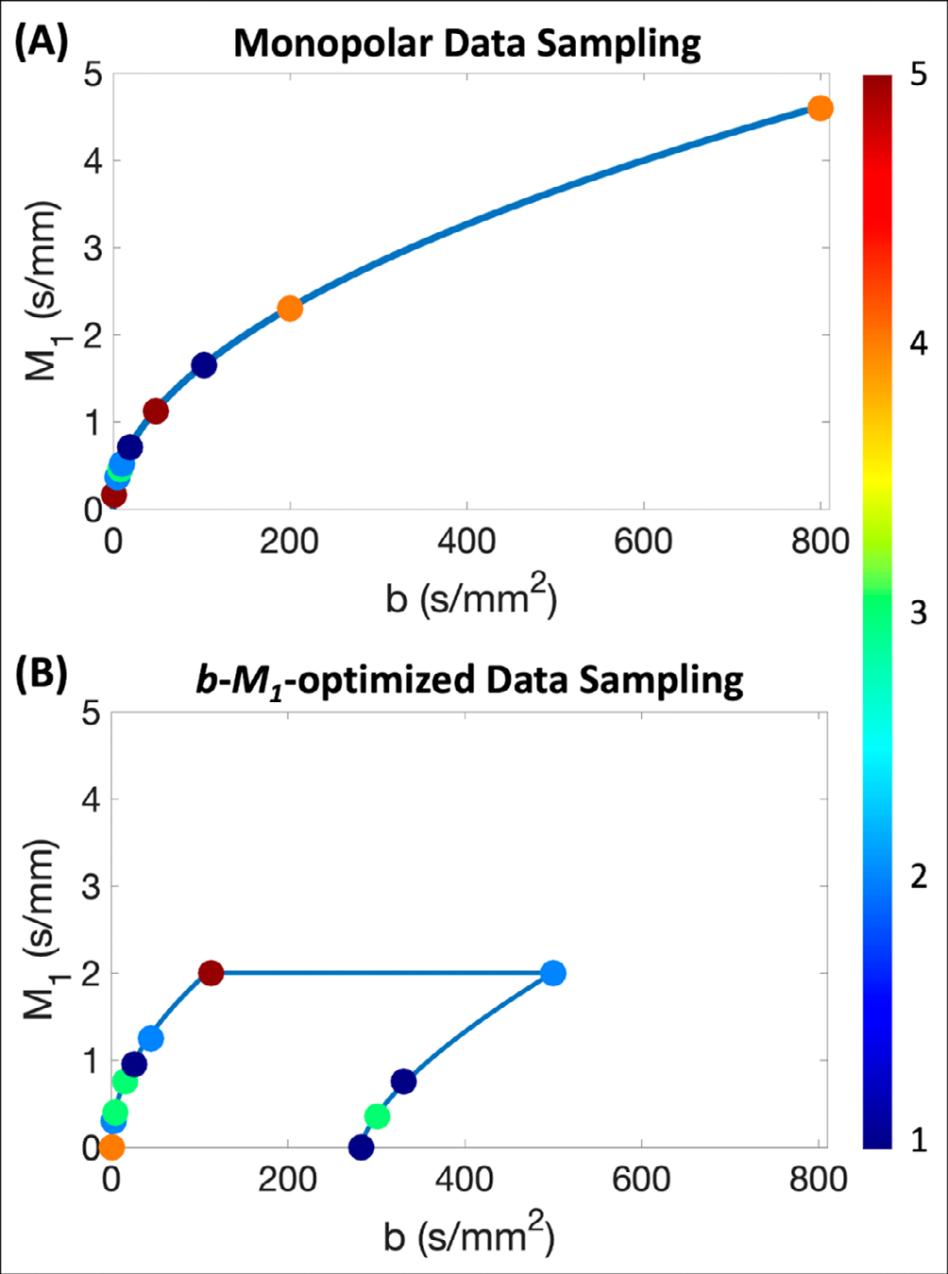
(A) Monopolar and (B) *b*‐value and first‐order motion moment [*M*_1_] optimized IVIM data samplings. Each *b*‐*M*_1_ pair is color coded based on number of repetitions (see Table 2). For the monopolar sampling, *b*‐*M*_1_ pairs were constrained to a curve (blue line) determined for monopolar gradient waveforms with *b*_max_ = 800 s/mm^2^ (designed for 3 T Signa Premier, GE Healthcare, Waukesha, WI). The *b*‐*M*_1_‐optimized sampling was constrained to *b*‐*M*_1_ pairs achievable within TE = 61.7 ms (i.e., TE required for *b* = 500 s/mm^2^ and *M*_1_ = 2.00 s/mm) and *M*_1_ ≤ 2.00 s/mm for motion‐robustness (area enclosed by blue lines).

For each flow rate, two IVIM acquisitions were obtained with identical slice locations using the parameters given in Table 2: (1) *b*‐value optimized IVIM with conventional monopolar gradient waveforms (optimized using previously published Cramer‐Rao lower bound‐based procedures [8, 15, 17]) and (2) a recently proposed *b*‐value and first‐order motion moment (*M*_1_) optimized IVIM acquisition [17] (Figure 2). For the *b*‐*M*_1_‐optimized acquisition, identical *b*‐*M*_1_‐pairs were used as Simchick et al. [17] However, the diffusion and flow encoding gradient waveforms were slightly modified to include direct contributions of imaging gradients on *b* and *M*_1_. Both monopolar and *b*‐*M*_1_‐optimized acquisitions were obtained to evaluate pseudo‐diffusion and ballistic IVIM signal models, respectively (see Section 2.4.4). For flow = 0_end_, the monopolar acquisition was not obtained due to logistical time constraints (e.g., MRI system access and scheduling constraints).

**TABLE 2:** Intravoxel incoherent motion (IVIM) acquisition parameters.

	Monopolar									*b*‐*M*_1_‐optimized										
*b* (s/mm^2^)	1	5	8	10	19	48	103	200	800	1	3	5	16	26	45	113	283	301	331	500
*M*_1_ (s/mm)	0.18	0.38	0.48	0.53	0.73	1.14	1.67	2.32	4.61	0	0.30	0.40	0.75	0.95	1.25	2.00	0	0.35	0.75	2.00
Repetitions (sum = 27)	5	2	3	2	1	5	1	4	4	4	2	3	3	1	2	5	1	3	1	2
TE (ms)	50.5									61.7										
TR (ms)	3000																			
Diffusion Directions	3 (anterior–posterior, right–left, superior–inferior)																			
FOV	28.8 × 28.8 cm^2^ (coronal plane)																			
In‐plane resolution	4.5 × 4.5 mm (64 × 64 matrix)																			
Slice thickness	5 mm (18–22 slices; no gap)																			
Readout bandwidth	±250 kHz (encoding direction: superior–inferior)																			
Acceleration	phase = 2; slice = 1																			
Fat suppression	Spatial‐spectral excitation																			
Acquisition time	4:06 min																			

### 2.4 MR Data Analysis

#### 2.4.1 Preservation Solution Analysis

Parameter maps of *R*_1ps_, *R*_2ps_, and *D*_ps_ were obtained using non‐linear least squares fitting of the multi‐TI fast‐spin‐echo, multi‐TE spin‐echo, and multi‐*b* monopolar IVIM datasets to the following equations, respectively

(1) $$S\left(\mathrm{TI}\right)=\left|S_{0}\left(1-2ke^{-R_{1\mathrm{ps}}\mathrm{TI}}\right)\right|$$

(2) $$S\left(\mathrm{TE}\right)=S_{0}e^{-R_{2\mathrm{ps}}\mathrm{TE}}$$

(3) $$S\left(b\right)=S_{0}e^{-bD_{\mathrm{ps}}}$$

where *k* in Equation (1) is the inversion pulse efficiency. *S*_0_ from each equation, as well as *k*, were free parameters and were also fit. Estimates of *R*_1ps_, *R*_2ps_, and *D*_ps_ were obtained by measuring the mean value from a circular ROI placed within each vial of preservation solution on a central slice. *R*_1ps_, *R*_2ps_, and *D*_ps_ estimates from vials collected before versus after each MRI exam were compared using paired sample *t*‐tests and reproducibility coefficients (=1.96*SD of differences).

#### 2.4.2 T1‐Weighted Liver Volume Estimation

All liver T1‐weighted datasets were interpolated to a matrix size of 512 × 512 (from 260 × 200) using in‐plane *k*‐space zero‐filling. For each explanted liver and total volumetric flow rate, an ROI of the entire liver was manually segmented by MR trainee JR with 4 years of liver imaging experience using the T1‐weighted images to estimate liver volume.

#### 2.4.3 Liver STEAM‐MRS Parameter Estimation

Using a previously published procedure [54], liver STEAM‐MRS data were fit using a 7‐peak (1 water and 6 fat) spectral model to simultaneously obtain separate water and fat estimates of *R*_1_ and *R*_2_, as well as proton‐density fat fraction. Water *R*_1_ and *R*_2_ were of particular interest in this work and were used to represent *R*_1_ and *R*_2_ of tissue (*R*_1t_ and *R*_2t_, respectively) when performing relaxation correction (see Section 2.4.5).

#### 2.4.4 Liver IVIM Parameter Estimation

All liver IVIM datasets were interpolated to a matrix size of 256 × 256 (from 96 × 96) using in‐plane *k*‐space zero‐filling. IVIM parameter maps of the diffusion coefficient (*D*), perfusion signal fraction (*F*) (i.e., signal fraction of incoherently flowing spins), pseudo‐diffusion coefficient (D*), and/or blood velocity standard deviation (*V*_b_) were obtained for each IVIM dataset through voxelwise fitting of the pseudo‐diffusion (Equation 4) and/or ballistic (Equation 5) IVIM signal models [13]

(4) $$S\left(b\right)=S_{0}\left[\left(1-F\right)e^{-\mathrm{bD}}+Fe^{-bD^{*}}\right]$$

(5) $$S\left(b,M_{1}\right)=S_{0}\left[\left(1-F\right)e^{-\mathrm{bD}}+Fe^{-bD_{\mathrm{ps}}}e^{-M_{1}^{2}V_{b}^{2}/2}\right]$$

where *D*_ps_ was fixed to 1.75 × 10^−3^ mm^2^/s based on preservation solution analysis (see Section 3.3.1). Monopolar IVIM datasets were fit to Equations (4) and (5) using segmented (two‐step) fitting to enhance fitting stability. First, *D* was estimated using *b* ≥ 200 s/mm^2^ data (assuming the incoherent flow component of the IVIM signal was saturated) and linear least squares fitting. Then, *S*_0_, *F*, and D* or *V*_b_ were estimated using the previously determined *D* estimate and nonlinear least squares fitting of all acquired data. Due to the IVIM signal's dependence on *M*_1_, the *b*‐*M*_1_‐optimized datasets were not expected to exhibit smooth bi‐exponential decay as a function of *b*; therefore, *b*‐*M*_1_‐optimized datasets were only fit to Equation (5). Further, the incoherent flow component of the IVIM signal was not expected to be saturated for all data with *b* ≥ 200 s/mm^2^ for the *b*‐*M*_1_‐optimized datasets; therefore, full (one‐step) nonlinear least squares fitting was performed.

For each explanted liver, a 3D ROI of the entire liver, including all slices, was manually segmented by MR scientist GS with 9y of liver imaging experience using *b* = 1 s/mm^2^ images from the monopolar datasets. Major vessels and other non‐parenchymal structures were then removed from the ROI using a previously described Blood Velocity standard deviation Distribution fitting method [8, 19], which is based on observed multi‐modal spatial distributions of *V*_*b*_ in the liver. From this multi‐modal distribution, the liver was separated into three distinct spatial locations: Ω_1_ (slow microvascular flow), Ω_2_ (fast microvascular flow), and Ω_3_ (major vessels and voxels with unstable fitting, excluded from further analysis). Median estimates of *D*, *F*, *V*_*b*_, and/or D* were determined for each corresponding parenchymal ROI (i.e., within Ω_1_ and Ω_2_) and flow rate.

#### 2.4.5 Relaxation Correction of Perfusion Signal Fraction

Estimates of *F* were corrected for confounding effects of *R*_1_ (=1/*T*_1_) and *R*_2_ (=1/*T*_2_) relaxation [8, 18, 55, 56, 57] using the following analytical equation

(6) $$F_{c}=\frac{Fe^{R_{2\mathrm{ps}}\mathrm{TE}}{\left(1-e^{-R_{1\mathrm{ps}}\mathrm{TR}}\right)}^{-1}}{Fe^{R_{2\mathrm{ps}}\mathrm{TE}}{\left(1-e^{-R_{1\mathrm{ps}}\mathrm{TR}}\right)}^{-1}+\left(1-F\right)e^{R_{2t}\mathrm{TE}}{\left(1-e^{-R_{1t}\mathrm{TR}}\right)}^{-1}}$$

For each explanted liver and flow rate, *R*_1t_ and *R*_2t_ obtained from STEAM‐MRS and mean *R*_1ps_ and *R*_2ps_ across the vials collected before/after each MRI exam were used when performing relaxation correction. In cases where vials were not collected before/after each MRI exam, mean *R*_1ps_ and *R*_2ps_ across vials from all livers were used when performing relaxation correction.

#### 2.4.6 Liver Volume, STEAM‐MRS, and IVIM Statistical Analysis

For liver volume and each estimated STEAM‐MRS and IVIM parameter, linear mixed‐effects modeling was performed, including four fixed and one random effect: (1) fixed binary effect of applied flow (i.e., 0 for no applied flow and 1 for applied flow), (2) fixed effect of total volumetric flow rate, (3) fixed temporal effect, (4) fixed binary effect of moderate‐to‐advanced fibrosis (i.e., 0 for fibrosis stages F0‐1 and 1 for F2‐4), and (5) random effect of explanted liver differences. The fixed temporal effect was included to evaluate changes or effect(s) in the estimated parameters over time that were unrelated to flow and/or indirectly related to flow, such as accumulation of preservation solution in the tissue over time. Since the flow rates were applied sequentially during data acquisition, this effect could only be evaluated in cases where flow = 0_start_ and 0_end_ data were acquired (i.e., for *b*‐*M*_1_‐optimized IVIM and STEAM‐MRS data), except for estimates of *V*_*b*_ which could not be accurately estimated when flow = 0 and *F*_*c*_ = 0. Otherwise, this effect was omitted from the mixed‐effects model (i.e., for *V*_*b*_ and all monopolar IVIM estimates). Assuming accumulation of preservation solution in the tissue was the primary contribution to the temporal effect and no accumulation occurred during flow = 0, the temporal effect was modeled assuming discrete timepoints of *t* = 0, 1/3, 2/3, 1, and 1 a.u. corresponding to flow rates of 0_start_, 0.6, 0.9, 1.2, and 0_end_ L/min, respectively. For each estimated parameter, *F*‐tests were used to determine if each fixed effect was significant across all explanted livers, and effect sizes were determined.

If the temporal effect(s) was determined to be significant for a given parameter, correction of the individual parameter estimates for this effect was performed. Similar to above, a linear effect of time was assumed with discrete timepoints of *t* = 1/3, 2/3, and 1 a.u. corresponding to flow rates of 0.6, 0.9, and 1.2 L/min, respectively, and with an effect size equal to the difference between flow = 0_start_ and 0_end_ estimates. Corrected estimates were obtained by subtracting 1/3, 2/3, and 1 of the difference from the 0.6, 0.9, and 1.2 L/min estimates, respectively. In cases where T1‐weighted, STEAM‐MRS, and/or *b*‐*M*_1_‐optimized IVIM data were not acquired for flow = 0_end_ due to time constraints, the mean difference between flow = 0_start_ and 0_end_ estimates across all livers was used when performing correction for temporal effect(s). Then, linear mixed‐effects analysis was performed as described above using the corrected estimates and the model omitting the temporal effect. Additionally, paired sample *t*‐tests and Bland–Altman analysis were performed using flow = 0_start_ and 0_end_ liver volume, STEAM‐MRS, and IVIM estimates.

## 3 Results

### 3.1 Donors and Explanted Livers

Eight (*n* = 8) explanted livers from deceased donors (3 M/5 F; age = 49–69 years; BMI = 25.6–46.4 kg/m^2^) and with various fibrosis stages (Table 1) were obtained and successfully imaged within 22.5–81.3 h of preservation. For one (*n* = 1) explanted liver, ultrasonic flowmeter measurements of venous and arterial flow rates were not obtained. For one (*n* = 1), one (*n* = 1), and two (*n* = 2) explanted livers, the T1‐weighted, *b*‐*M*_1_‐optimized IVIM, and STEAM‐MRS acquisitions, respectively, were not acquired with flow = 0_end_ due to time constraints. For one (*n* = 1) explanted liver, a vial of preservation solution was only collected after the MRI exam. For two (*n* = 2) explanted livers, vials of preservation solution were not collected before or after the MRI exam.

In the interest of reproducible research, data that support the findings of this study are available upon request and data use agreement at 10.5281/zenodo.17536166. All individual measurements are also provided in Supporting Information.

### 3.2 Perfusion Setup

Using the ultrasonic flowmeter, total volumetric flow rates = 0.60 ± 0.01, 0.90 ± 0.02, and 1.21 ± 0.03 L/min were observed for target flow rates = 0.6, 0.9, and 1.2 L/min, respectively. Venous flow rates = 0.48 ± 0.08, 0.69 ± 0.12, and 0.90 ± 0.15 L/min and arterial flow rates = 0.12 ± 0.07, 0.21 ± 0.11, and 0.31 ± 0.13 were also observed, respectively. This resulted in venous‐to‐arterial ratios = 5.17 ± 2.89, 4.28 ± 2.32, and 3.55 ± 1.75, respectively, with significantly lower (*p* ≤ 0.02) ratios observed for flow = 1.2 L/min compared to flow = 0.6 and 0.9 L/min. Comparing F0‐1 and F2‐4 livers, no significant differences (*p* ≥ 0.34) were observed in the venous‐to‐arterial ratios.

### 3.3 MR Data Analysis

#### 3.3.1 Preservation Solution Analysis

Similar estimates of *R*_1ps_, *R*_2ps_, and *D*_ps_ were observed for vials of preservation solution collected before (*R*_1ps_ = 0.57 ± 0.16 s^−1^, *R*_2ps_ = 3.05 ± 1.19 s^−1^, and *D*_ps_ = 1.78 ± 0.05 × 10^−3^ mm^2^/s) and after (*R*_1ps_ = 0.57 ± 0.13 s^−1^, *R*_2ps_ = 3.19 ± 1.19 s^−1^, and *D*_ps_ = 1.77 ± 0.06 × 10^−3^ mm^2^/s) the MRI exams. No significant differences (*p* ≥ 0.59) and reproducibility coefficients = 0.08 s^−1^, 1.24 s^−1^, and 0.09 × 10^−3^ mm^2^/s for *R*_1ps_, *R*_2ps_, and *D*_ps_, respectively, were observed.

#### 3.3.2 T1‐Weighted Liver Volume Analysis

**FIGURE 3:**
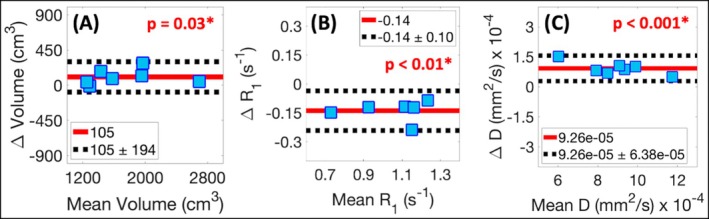
Temporal effect(s) unrelated to flow or indirectly related to flow, such accumulation of the preservation solution in the tissue over time, may have affected specific quantitative parameters. Shown are Bland–Altman plots of (A) liver volume, (B) water *R*_1_ obtained from STEAM‐MRS, and (C) *D* obtained from the *b*‐*M*_1_‐optimized IVIM acquisition for the flow = 0 estimates obtained at the start (0_start_) versus end (0_end_) of the MRI exams. Significantly (*p* < 0.05; denoted by *) lower *R*_1_ (*p* < 0.01; mean differences = −0.14 s^−1^) and higher volumes (*p* = 0.03; mean difference = 105 cm^3^) and *D* (*p* < 0.001; mean difference = 0.93 × 10^−4^ mm^2^/s) were observed at the end of the exams.

Averages and SDs of liver volume estimates across all explanted livers for each total volumetric flow rate are provided in Table 3. Using mixed‐effects analysis, no significant (*p* = 0.69) effect of applied flow was observed for volume (Table 4). However, significant (*p* ≤ 0.02) flow rate and temporal effects were observed for volume with effect sizes = 156 cm^3^ per L/min and 117 cm^3^, respectively. Further, significantly higher (*p* = 0.03) volume estimates were observed at flow = 0_end_ versus 0_start_ (mean difference = 105 cm^3^) (Figure 3A). Correcting for the temporal effect on volume (i.e., volume_c_), a significant (*p* < 0.01) effect of flow rate was observed with an effect size = 173 cm^3^ per L/min, whereas no significant (*p* = 0.55) effect of applied flow was observed. Comparing F0‐1 and F2‐4 livers, no significant differences (*p* ≥ 0.45) were observed for volume or volume_c_.

**TABLE 3:** Liver volume, STEAM‐MRS, and IVIM estimates.

	Total volumetric flow rate (L/min)				
	0_start_	0.6	0.9	1.2	0_end_
T1‐weighted					
Volume (cm^3^)	1687 ± 505	1799 ± 525	1845 ± 531	1945 ± 555	1792 ± 523
Volume_c_ (cm^3^)	—	1764 ± 522	1767 ± 531	1849 ± 536	—
STEAM‐MRS					
*R*_1_ (s^−1^)	1.10 ± 0.17	1.02 ± 0.21	0.99 ± 0.20	0.96 ± 0.19	0.98 ± 0.19
*R*_1c_ (s^−1^)	—	1.07 ± 0.21	1.09 ± 0.20	1.10 ± 0.18	—
*R*_2_ (s^−1^)	35.6 ± 13.3	34.7 ± 15.8	32.0 ± 15.2	31.8 ± 14.5	36.1 ± 15.0
*PDFF* (%)	8.16 ± 8.52	8.51 ± 9.30	7.78 ± 8.43	8.06 ± 8.81	8.41 ± 12.8
IVIM: Monopolar					
*D* (×10^−3^ mm^2^/s)	0.67 ± 0.16	0.72 ± 0.15	0.73 ± 0.15	0.74 ± 0.15	—
*F* (%)	1.82 ± 1.31	8.85 ± 6.59	9.23 ± 6.39	9.12 ± 6.19	—
*F*_*c*_ (%)	0.42 ± 0.27	2.12 ± 0.85	2.35 ± 0.77	2.39 ± 0.72	—
*V*_*b*_ (mm/s)	—	2.13 ± 0.48	2.40 ± 0.60	2.54 ± 0.68	—
D* (×10^−3^ mm^2^/s)	—	34.2 ± 14.4	44.2 ± 21.0	49.6 ± 24.4	—
IVIM: *b*‐*M*_1_‐optimized					
*D* (×10^−3^ mm^2^/s)	0.85 ± 0.18	0.94 ± 0.16	0.97 ± 0.15	0.99 ± 0.14	0.94 ± 0.16
*D*_*c*_ (×10^−3^ mm^2^/s)	—	0.91 ± 0.16	0.91 ± 0.16	0.90 ± 0.16	—
*F* (%)	−0.16 ± 0.79	9.09 ± 7.81	9.69 ± 7.41	9.48 ± 6.87	0.50 ± 0.91
*F*_*c*_ (%)	−0.12 ± 0.24	1.52 ± 0.82	1.78 ± 0.85	1.82 ± 0.86	0.03 ± 0.08
*V*_*b*_ (mm/s)	—	3.12 ± 0.87	3.48 ± 0.84	3.76 ± 0.75	—

*Note*: Values are averages ± SDs across all explanted livers. Note that *F*_*c*_ corresponds to relaxation‐corrected perfusion signal fraction, whereas volume_c_, *R*_1c_, and *D*_*c*_ correspond to temporal effect corrected volume, *R*_1_, and diffusion coefficient (see Sections 2.4.5 and 2.4.6 for details).

**TABLE 4:** Mixed‐effects analysis.

	Flow rate		Applied flow		Temporal		Fibrosis (F0‐1 vs F2‐4)		Model fit
	*p*	Effect size (units/[L/min])	*p*	Effect size (units/a.u.)	*p*	Effect size (units/a.u.)	*p*	Effect size (units/a.u.)	*R* ^2^
T1‐weighted									
Volume (cm^3^)	0.02*	156	0.69	−24	< 0.01*	117	0.45	268	0.99
Volume_c_ (cm^3^)	< 0.01*	173	0.55	−31	—	—	0.49	246	0.99
STEAM‐MRS									
*R*_1_ (s^−1^)	0.66	0.02	0.27	−0.05	< 0.001*	−0.12	0.60	−0.06	0.94
*R*_1c_ (s^−1^)	0.26	0.04	0.13	−0.05	—	—	0.69	−0.05	0.96
*R*_2_ (s^−1^)	0.26	−2.94	0.68	0.97	0.21	−1.75	0.48	6.59	0.97
PDFF (%)	0.25	−1.65	0.52	0.81	0.25	0.87	0.70	2.37	0.98
IVIM: Monopolar									
*D* (mm^2^/s)	0.39	0.41 × 10^−4^	0.60	0.25 × 10^−4^	—	—	0.95	0.06 × 10^−4^	0.86
*F*_*c*_ (%)	0.15	0.46	< 0.001*	1.46	—	—	0.14	−0.52	0.88
*V*_*b*_ (mm/s)	0.01*	0.69	< 0.001*	1.74	—	—	< 0.01*	−0.59	0.93
D* (mm^2^/s)	0.01*	25.7 × 10^−3^	0.04*	19.6 × 10^−3^	—	—	< 0.01*	−19.8 × 10^−3^	0.81
IVIM: *b*‐*M*_1_‐optimized									
*D* (mm^2^/s)	0.89	0.07 × 10^−4^	0.30	0.51 × 10^−4^	< 0.01*	0.85 × 10^−4^	0.81	0.23 × 10^−4^	0.89
*D*_*c*_ (mm^2^/s)	0.97	−0.01 × 10^−4^	0.20	0.54 × 10^−4^	—	—	0.98	0.03 × 10^−4^	0.92
*F*_*c*_ (%)	0.46	0.33	< 0.01*	1.41	0.48	0.16	< 0.01*	−0.74	0.81
*V*_*b*_ (mm/s)	0.01*	1.06	< 0.001*	2.48	—	—	0.01*	−0.61	0.94

*Note*: Since flow rates were applied sequentially during data acquisition, the temporal effect could not be evaluated in cases where flow = 0_start_ and 0_end_ data wasn't acquired (i.e., for the monopolar IVIM data) or for estimates of *V*_*b*_ which could not be accurately estimated when flow = 0 and *F*_*c*_ = 0. If the temporal effect was determined to be significant (*p* < 0.05; denoted by *) for a given parameter, correction of the individual parameter estimates for this effect was performed (see Section 2.4.6 for details), and mixed‐effects analysis was performed using the corrected estimates (i.e., volume_c_, *R*_1c_, and *D*_*c*_) while omitting the temporal effect from the model. Note that *F*_*c*_ corresponds to relaxation‐corrected perfusion signal fraction (see Sections 2.4.5 for details).

#### 3.3.3 STEAM‐MRS Analysis

Averages and SDs of STEAM‐MRS estimates across all explanted livers for each total volumetric flow rate are provided in Table 3. Using mixed‐effects analysis, no significant (*p* ≥ 0.21) effect of flow rate, applied flow, or temporal effect(s) was observed for water *R*_2_ or proton‐density fat fraction (Table 4). Similarly, no significant differences (*p* ≥ 0.16) were observed for these parameters when comparing flow = 0_start_ and 0_end_ estimates. Further, no significant (*p* ≥ 0.27) effect of flow rate or applied flow was observed in water *R*_1_ estimates. However, a significant (*p* < 0.001) temporal effect was observed for water *R*_1_ with an effect size = −0.12 s^−1^, and significantly lower (*p* < 0.01) *R*_1_ estimates were observed at flow = 0_end_ versus 0_start_ (mean difference = −0.14 s^−1^) (Figure 3B). Correcting for the temporal effect on *R*_1_ (i.e., *R*_1c_), no significant (*p* ≥ 0.13) effect of flow rate or applied flow was observed. Statistical analysis of fat *R*_1_ and *R*_2_ estimates was not performed due to low proton‐density fat fraction (≤ 5.4%) in most (6 out of 8) of the explanted livers. Comparing F0‐1 and F2‐4 livers, no significant differences (*p* ≥ 0.48) were observed for water *R*_1_, water *R*_2_, or proton‐density fat fraction.

#### 3.3.4 IVIM Analysis

**FIGURE 4:**
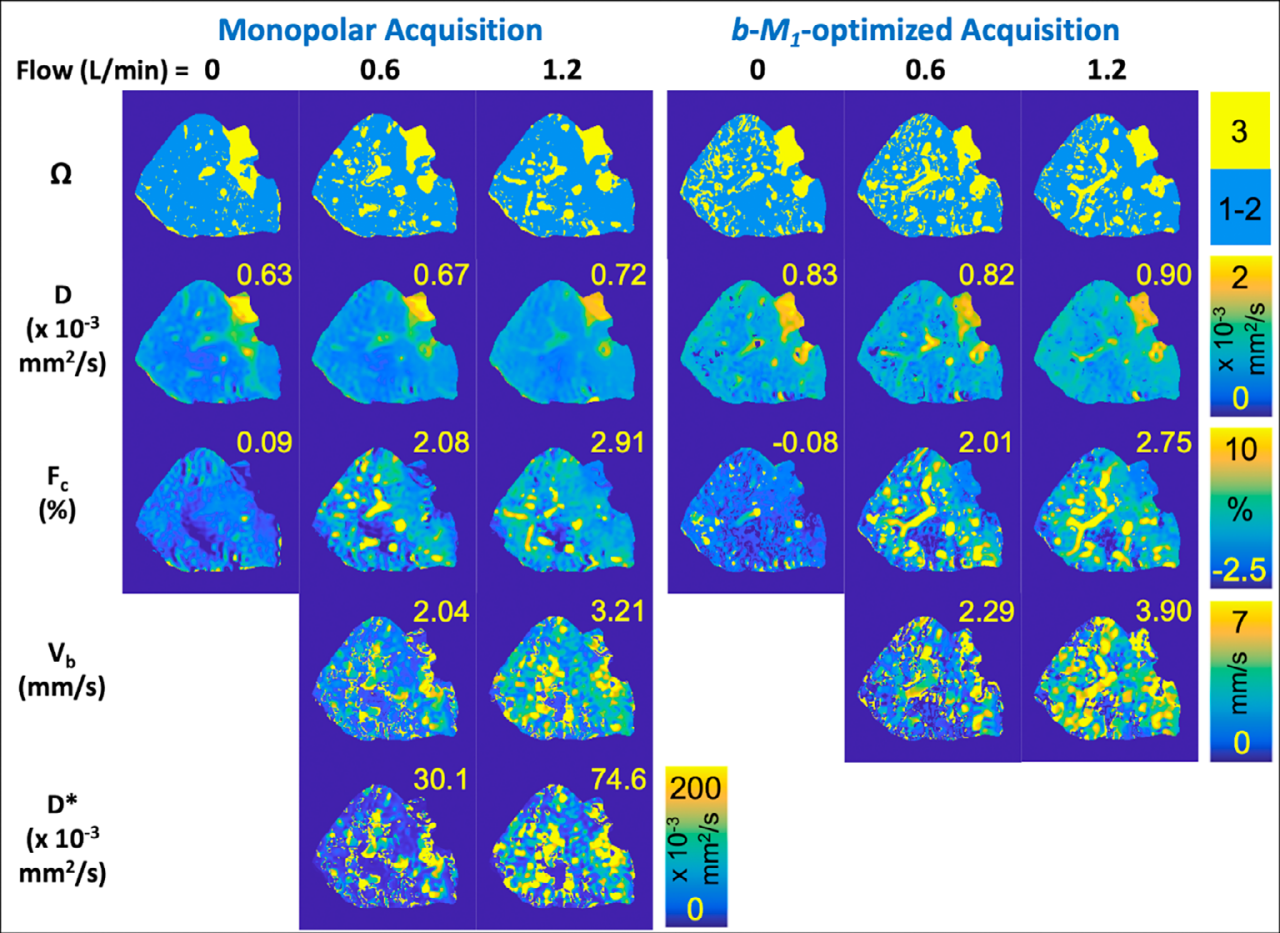
Representative explanted liver parameter maps of the diffusion coefficient (*D*), relaxation‐corrected perfusion signal fraction (*F*_*c*_), blood velocity standard deviation (*V*_*b*_), and pseudo‐diffusion coefficient (D*) obtained using monopolar and *b*‐*M*_1_‐optimized IVIM data acquisitions at different flow rates. Median estimates for each parameter, which are given in the upper right corner of each map, were obtained within the spatial locations Ω_1_ and Ω_2_ (top row). *V*_*b*_ and D* are not shown for flow = 0, as they cannot be accurately estimated when *F*_*c*_ is near zero.

**FIGURE 5:**
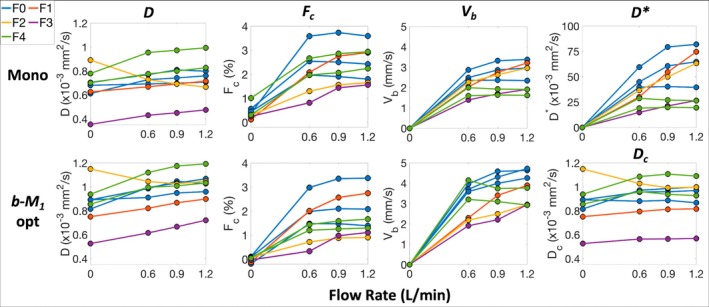
IVIM estimates demonstrated varying dependencies on total volumetric flow rate. Shown are line graphs where each line represents an explanted liver, and the line's color represents the liver's fibrosis stage (key in upper left corner). For both the monopolar (first row) and *b‐M*_1_‐optimized (second row) IVIM acquisitions, estimates of *D* (first column) increased over time (significant temporal effect (*p* < 0.01) and non‐significant flow rate effects (*p* ≥ 0.30)). Correcting for temporal effects, *D*_*c*_ (lower right corner) was relatively constant (*p* = 0.19 and 0.97 for applied flow and flow rate effects, respectively). Estimates of *F*_*c*_ (second column) were approximately zero for flow = 0 and a relatively constant, positive, non‐negligible value with flow (*p* < 0.01 and ≥ 0.15 for applied flow and flow rate effects, respectively). Estimates of *V*_*b*_ (third column) and D* (upper right corner) increased with flow rate (*p* ≤ 0.04 for applied flow and flow rate effects).

Representative IVIM parameter maps of *D*, *F*_*c*_, *V*_*b*_, and D* at different flow rates for the monopolar and *b*‐*M*_1_‐optimized IVIM acquisitions are provided in Figure 4. Similarly, averages and SDs of IVIM estimates across all explanted livers for each flow rate and acquisition are provided in Table 3. For both the monopolar and *b*‐*M*_1_‐optimized acquisitions, *D* increased over time; *F* was approximately zero for flow = 0 and a relatively constant, positive, non‐negligible value for flow > 0; *V*_*b*_ and D* increased with flow rate, except for livers with F4 which had decreasing *V*_*b*_ and D* across the non‐zero flow rates (Figure 5).

Using mixed‐effects analysis, no significant (*p* ≥ 0.30) effect of flow rate or applied flow was observed for estimates of *D* obtained using either the monopolar or *b*‐*M*_1_‐optimized IVIM acquisition (Table 4). However, for the *b*‐*M*_1_‐optimized acquisition, a significant (*p* < 0.01) temporal effect was observed for *D* with an effect size = 0.85 × 10^−4^ mm^2^/s, and significantly larger (*p* < 0.001) *D* estimates were observed at flow = 0_end_ versus 0_start_ (mean difference = 0.93 × 10^−4^ mm^2^/s) (Figure 3C). Correcting for the temporal effect on *D* (i.e., *D*_*c*_), no significant (*p* ≥ 0.20) effect of flow rate or applied flow was observed, and estimates of *D*_*c*_ were relatively constant (Table 3) (Figure 5). For both acquisitions, a significant (*p* < 0.01) effect of applied flow was observed for estimates of *F*_*c*_ with effect sizes = 1.46 and 1.41% for monopolar and *b*‐*M*_1_‐optimized acquisitions, respectively, whereas no significant (*p* ≥ 0.15) effect of flow rate was observed. For estimates of *V*_*b*_, significant effects (*p* ≤ 0.01) of applied flow and flow rate were observed (applied flow effect size = 1.74 and 2.48 mm/s for monopolar and *b*‐*M*_1_‐optimized acquisitions, respectively, and flow rate effect size = 0.69 and 1.06 mm/s per L/min, respectively). Similarly, significant effects (*p* ≤ 0.04) of applied flow and flow rate were also observed for estimates of D* obtained for the monopolar acquisition (applied flow effect size = 19.6 × 10^−3^ mm^2^/s and flow rate effect size = 25.7 × 10^−3^ mm^2^/s per L/min).

**FIGURE 6:**
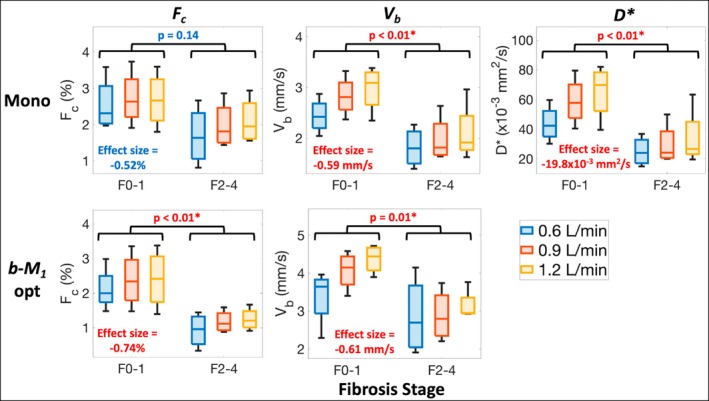
IVIM has the potential to differentiate livers with no‐to‐mild fibrosis (stages ≤ 1) from moderate‐to‐advanced fibrosis (stages ≥ 2). Shown are boxplots of *F*_*c*_ (first column), *V*_*b*_ (second column), and D* (third column) obtained using the monopolar (first row) and *b‐M*_1_‐optimized (second row) IVIM acquisitions for explanted livers with fibrosis stages F0‐1 and F2‐4 perfused with various total volumetric flow rates (key in the bottom right). Based on mixed‐effects analysis (Table 4), significantly (*p* < 0.05; denoted by *) lower *F*_*c*_, *V*_*b*_, and D* estimates were observed for the F2‐4 livers (*p* ≤ 0.01) (effect sizes = −0.74%, −0.61 to −0.59 mm/s, and −19.8 × 10^−3^ mm^2^/s, respectively), except for *F*_*c*_ obtained for the monopolar acquisition (*p* = 0.14).

Comparing F0‐1 and F2‐4 livers, significantly lower (*p* ≤ 0.01) *F*_*c*_, *V*_*b*_, and D* estimates were observed for F2‐4 livers (effect sizes = −0.74%, −0.61 to −0.59 mm/s, and −19.8 × 10^−3^ mm^2^/s, respectively), except for *F*_*c*_ estimates obtained for the monopolar acquisition (*p* = 0.14) (Figure 6). No significant differences (*p* ≥ 0.81) were observed in estimates of *D* or *D*_*c*_.

## 4 Discussion

In this work, the feasibility of using perfused explanted human livers for validating IVIM was demonstrated. Importantly, the perfused explanted livers served as biologically accurate systems with distinct microstructural and microvascular flow compartments (i.e., diffusion and incoherent flow components of the IVIM signal were independent and dependent on flow, respectively). The diffusion coefficient (*D*) was independent of applied flow and total volumetric flow rate; the relaxation‐corrected perfusion signal fraction (*F*_*c*_) was approximately zero when no flow was applied and a positive, non‐negligible value that was independent of flow rate with applied flow; blood velocity SD (*V*_*b*_) and pseudo‐diffusion coefficient (D*) were dependent on applied flow and flow rate. Further, the potential of IVIM to differentiate livers with no‐to‐mild fibrosis (stages ≤ F1) from moderate‐to‐advanced fibrosis (stages ≥ F2) was demonstrated using *F*_*c*_, *V*_*b*_, and D*. This result may be clinically relevant, as the risk of liver‐related mortality increases significantly for patients with stages ≥ F2 in the presence of steatotic liver disease [58]. Various treatments, including the recently Food and Drug Administration‐approved drug resmetirom, are usually recommended for these patients based on prior authorization criteria of fibrosis stage [59, 60].

Several studies have used IVIM to evaluate liver fibrosis [1, 2, 3, 4, 5, 6, 7], hepatitis [61], and fatty liver disease in vivo [62, 63]. However, in vivo validation is challenging due to physiologic motion, unknown vascular flow conditions, and core biopsy variability. Alternatively, perfused explanted liver MRI experiments, as demonstrated in this work, can be performed in a highly controlled setting with precise inflow and without confounding effects of respiratory and cardiac motion. Further, for explanted livers specifically available for research purposes, multiple wedge biopsies can be obtained throughout the liver to avoid sampling bias and variability associated with smaller core biopsies [22, 23, 24, 25]. Evaluating perfused explanted livers with a wide range of disease may enable better characterization of IVIM parameter relationships with histologic data.

Perfused explanted liver studies may also help with the development of accurate IVIM signal models and reproducible quantitative IVIM methods. In the liver, the IVIM signal has been shown to depend on several acquisition parameters, including *b*, *M*_1_, *TE*, and encoding duration [8, 14, 18, 55, 56, 57, 64]. Several IVIM signal models have been proposed for different flow regimes, including pseudo‐diffusion (*b*‐value only), ballistic (*b* and *M*_1_ dependent) [13, 19], and intermediate (*b* and encoding duration dependent) [14, 65, 66] regime models. The flow regime and appropriate IVIM signal model may also vary spatially throughout the liver [19, 67]. Perfused explanted liver studies with highly controlled inflow may help better characterize IVIM signal behavior, including the evaluation of temporal, spatial, and acquisition‐related IVIM signal dependencies, while avoiding confounding factors such as physiologic motion. Further, by modulating the relaxation properties of the preservation solution (e.g., by adding gadolinium‐based contrast agents), relaxation correction methods and simultaneous multi‐slice IVIM methods with short TRs may be evaluated and validated [8, 17, 18, 55, 68, 69].

The proposed perfusion setup, including quantitative evaluation of microstructure and microvascular flow using IVIM, may also prove useful for evaluation of liver organ condition for transplantation. Current methods for evaluating organ condition include visual appearance, the donor's medical history, blood tests, core biopsies, and CT imaging. However, these methods are unable to directly probe microvascular flow, which may be damaged during the organ donation and preservation process. Additional quantitative MRI techniques, such as MR elastography for evaluation of fibroinflammation and chemical shift‐encoded MRI for evaluation of steatosis and iron overload, may be combined with IVIM to create a comprehensive MRI protocol to better identify organs that are suitable for transplantation, enabling efficient and life‐saving workflows in transplantation clinics. Further, ex vivo machine perfusion is an emerging clinical technology for preservation of explanted livers for transplantation. However, little is known about the quality and dynamics of liver perfusion in ex vivo settings. IVIM has the potential to provide insights into microvascular dynamics during ex vivo perfusion and help optimize machine perfusion techniques.

When designing IVIM phantoms or systems for validation, ideally the microstructural compartment or diffusion component (i.e., *D*) and the intravoxel ratio of flowing versus non‐flowing spins (i.e., *F* or *F*_*c*_) should be independent of externally controlled flow conditions, such as flow rate, velocity, or pressure. However, previous IVIM phantom designs lacked this independence [29, 30, 31, 32, 33], possibly due to fluid exchange between compartments and/or contributions of coherent flow on the MR signal. Using the perfused explanted liver system proposed in this work, *D* was independent of flow rate; however, a significant temporal effect(s) was observed. It was hypothesized that the temporal effect on *D* was primarily due to the accumulation of preservation solution in the tissue over time, as the explanted livers increased in volume during the MRI exams. A method for correcting estimates of *D* for temporal effect(s) was proposed, and *D*_*c*_ estimates were relatively constant and independent of the applied flow and flow rate with small residual effect sizes (applied flow effect size = 0.54 × 10^−4^ mm^2^/s and flow rate effect size = −0.01 × 10^−4^ mm^2^/s per L/min).

Accumulation of preservation solution in the tissue could also have resulted in smaller estimates of *F*_*c*_ over time due to an increase in the number of non‐flowing spins in the microstructural compartment contributing to the diffusion component. The increase in liver volume (due to solution accumulation and flow rate effect) may also have altered microvasculature geometry and the intravoxel microvascular volume, which may have affected estimates of *F*_*c*_, *V*_*b*_, and/or D*. However, in this work, estimates of *F*_*c*_ were observed to be independent of flow rate, suggesting that preservation solution accumulation and changes in liver volume did not significantly affect the microvasculature volume. Therefore, changes in liver volume due to the flow rate effect likely resulted from macrovasculature volume changes. Nevertheless, future studies evaluating possible macrovasculature volume changes are needed, and correction for temporal effect(s), such as solution accumulation, may be necessary for accurate evaluation of IVIM parameters.

In vivo, total hepatic blood flow ranges from approximately 0.8–1.5 L/min with venous‐to‐arterial ratios of approximately 3–4 [50, 52]. For the flow rate = 1.2 L/min used in this work, similar venous‐to‐arterial ratios = 3.55 ± 1.75 were observed. Further, the explanted liver estimates of *V*_*b*_ and D* obtained in this work using the *b*‐*M*_1_‐optimized and monopolar acquisitions, respectively (*V*_*b*_ = 3.76 ± 0.75 mm/s and D* = 49.6 ± 24.4 × 10^−3^ mm^2^/s), were comparable to previous in vivo studies using similar acquisitions (*V*_*b*_ = 3.23–5.22 mm/s and D* = 44.0–92.0 × 10^−3^ mm^2^/s) [1, 8, 15, 17, 64]. However, estimates of *D*_*c*_ and *F*_*c*_ obtained using the *b*‐*M*_1_‐optimized IVIM acquisition (*D*_*c*_ = 0.90 ± 0.16 × 10^−3^ mm^2^/s and *F*_*c*_ = 1.82% ± 0.86%) were smaller than in vivo liver estimates reported in previous studies using similar methods (*D =* 1.02–1.36 × 10^−3^ mm^2^/s and *F*_*c*_ = 6.43%–9.35%) [8, 15, 17, 64]. The observed difference in *D*_*c*_ was likely due to temperature effects, whereas the observed difference in *F*_*c*_ may have been due to differences between the preservation solution and blood (see next paragraph for further details).

Limitations of this work include several experimental versus in vivo differences, inability to store and reuse explanted livers in future studies, lack of advanced histology, assuming linear effects for mixed‐effects modeling, and a small sample size. Importantly, the perfused explanted liver experiments performed in this work used non‐pulsatile flow, non‐blood organ preservation solution, and varying venous‐to‐arterial ratios. Although the organ preservation solution used in this work had a similar diffusivity to that of blood [13, 64], differences in pulsatility and solution composition, specifically a lack of macromolecules and red blood cells, may have led to differences in the distribution of flow velocities in the microvasculature and differences in the density of flowing spins compared to in vivo. Differences in vascular resistance, which may have been affected by several factors, including vessel geometry, vessel stiffness, and liver stiffness, also likely contributed to the observed variability in the venous‐to‐arterial inflow ratios across livers and flow rates (SDs ≥ 1.75). In this study, the intrinsic resistance of each liver was allowed to dictate the venous‐to‐arterial inflows, resulting in micro‐ and macrovascular flow governed by the livers' tissue properties. This experimental design sought to avoid possible vasculature damage associated with artificial alteration of the resistance. Further, the experiments were performed at room temperature, and temperature effects on the IVIM parameters were not considered. Specifically, *D* has a strong dependence on temperature, which may have obscured possible differences between the F0‐1 and F2‐4 livers. Future studies will seek to independently control venous and arterial inflows, as well as monitor the liver and preservation solution temperatures throughout each MRI exam to correct for temperature effects.

Additionally, explanted livers cannot be preserved long‐term or be repeatedly perfused without risking tissue damage. Therefore, unlike IVIM phantoms constructed with packed microspheres or compressed sponges [29, 30, 31, 32], explanted livers cannot be stored and reused in future studies. Further, each explanted liver has unique vascular and tissue properties. Although it is possible to externally modulate flow conditions in a highly controlled manner using the proposed perfused explanted liver setup, directly modulating the liver's underlying tissue properties is not possible. Therefore, conducting long‐term studies and replicating experiments on an individual organ is not feasible. However, perfused explanted livers provide the opportunity to characterize the fundamental relationships between IVIM parameters and underlying tissue properties through histological validation. In this work, the fibrosis stage of each liver was obtained from clinical histology of core biopsies, and relaxation correction was performed based on estimates obtained from single‐voxel STEAM‐MRS, both of which may suffer from spatial sampling variability, particularly in fibrotic livers where the disease may occur homogeneously. Despite these limitations and the small sample size of only eight (*n* = 8) explanted livers, differences in the IVIM parameters were observed between F0‐1 and F2‐4 livers. However, this work lacks advanced histology evaluating features that more closely relate to microstructure and microvasculature, such as cellularity and vessel density [26, 27, 28]. Future studies, including the collection of histologic samples before and after experimentation, are needed to evaluate possible tissue damage resulting from preservation and/or the proposed perfusion setup.

Finally, when performing mixed‐effects modeling, the flow rate and temporal effects on the estimated STEAM‐MRS and IVIM parameters were assumed to be linear. Further, the temporal effect was modeled using discrete timepoints instead of continuous time. However, these effects may have been non‐linear and/or the data may not have been acquired equally spaced in time leading to bias in the determined effect sizes. Despite these limitations, the chosen mixed‐effects model fit the data well (*R* ^2^ ≥ 0.81).

## 5 Conclusion

Perfused explanted human livers from deceased donors may serve as biologically accurate systems for validation of quantitative IVIM techniques. Importantly, the proposed perfused explanted liver system contained distinct microstructural and microvascular flow compartments (i.e., diffusion and incoherent flow components of the IVIM signal were independent and dependent on flow, respectively).

## Conflicts of Interest

GE Healthcare provides research support to the University of Wisconsin. Unrelated to this project, Diego Hernando is co‐founder of Calimetrix LLC.

## Supporting information


**Table S1:** Individual measurements of estimated parameters obtained in this study.

## Data Availability

The data that support the findings of this study are available upon request and data use agreement at 10.5281/zenodo.17536166. All individual measurements of estimated parameters obtained in this study are also provided in Supporting Information.
